# Mechanisms of Neurodegeneration and Axonal Dysfunction in Progressive Multiple Sclerosis

**DOI:** 10.3390/biomedicines7010014

**Published:** 2019-02-20

**Authors:** Jorge Correale, Mariano Marrodan, María Célica Ysrraelit

**Affiliations:** Department of Neurology, FLENI, Buenos Aires 1428, Argentina; mmarrodan@fleni.org.ar (M.M.); mcysrraelit@fleni.org.ar (M.C.Y.)

**Keywords:** autoimmunity, axon, cortex, demyelination, mitochondria, multiple sclerosis, myelin, neurodegeneration, oligodendrocyte, progressive multiple sclerosis

## Abstract

Multiple Sclerosis (MS) is a major cause of neurological disability, which increases predominantly during disease progression as a result of cortical and grey matter structures involvement. The gradual accumulation of disability characteristic of the disease seems to also result from a different set of mechanisms, including in particular immune reactions confined to the Central Nervous System such as: (a) B-cell dysregulation, (b) CD8^+^ T cells causing demyelination or axonal/neuronal damage, and (c) microglial cell activation associated with neuritic transection found in cortical demyelinating lesions. Other potential drivers of neurodegeneration are generation of oxygen and nitrogen reactive species, and mitochondrial damage, inducing impaired energy production, and intra-axonal accumulation of Ca^2+^, which in turn activates a variety of catabolic enzymes ultimately leading to progressive proteolytic degradation of cytoskeleton proteins. Loss of axon energy provided by oligodendrocytes determines further axonal degeneration and neuronal loss. Clearly, these different mechanisms are not mutually exclusive and could act in combination. Given the multifactorial pathophysiology of progressive MS, many potential therapeutic targets could be investigated in the future. This remains however, an objective that has yet to be undertaken.

## 1. Introduction

Multiple Sclerosis (MS) is a chronic inflammatory disease of the Central Nervous System (CNS) leading to demyelination and diffuse neurodegeneration in both brain and spinal cord grey and white matter of the brain and spinal cord [1,2]. Although its etiology remains elusive results from immunological, genetic, and histopathology studies of patients with MS support the concept that autoimmunity plays a major role in disease pathogenesis [1,3]. Disease course can be highly variable, however most patients present recurring clinical symptoms from onset followed by total or partial recovery, the classic relapsing–remitting form of the disease (RRMS). After 10–15 years the pattern becomes progressive in up to 50% of untreated patients, and symptoms slowly progress over a period of many years. This stage is defined as secondary progressive MS (SPMS). Fifteen percent of MS patients can present a progressive from onset, and is named primary progressive MS (PPMS) [4]. Actually, it is not known to whether PPMS is a different form of MS or is simply SPMS, without identifiable clinical relapses.

The most characteristic brain tissue injury in MS is primary demyelination with partial preservation of axons [2]. In general, actively demyelinating plaques in RRMS involves the movement of immune cells from the periphery into the CNS, which is associated with disruption of the blood-brain-barrier (BBB). In contrast, progressive disease involves the development of compartmentalized pathological processes within the brain mediated mainly by resident CNS cells. Evidence of this comes from MRI showing decreased gadolinium (Gd) enhancement in CNS lesions found in progressive MS patients, indicating reduced BBB breakdown and less movement of immune cells into the CNS. Several tissue pathology findings are associated with progressive MS. The most prominent is brain atrophy, caused chiefly by degeneration and chronic demyelination of axons, ultimately leading to neuronal loss [5]. Representing a major cause of irreversible neurological disability [6]. Although imaging and neuropathological studies have shown that both axonal degeneration and neuronal death are present in acute or active MS lesions [7], progression likely occurs once axonal loss exceeds CNS compensatory capacity. Whether inflammation and neurodegeneration are primary or secondary processes, and how they interact during the course of disease remains unclear. Another major pathological substrate of progressive MS is cortical demyelination. Grey matter demyelination is also observed in cerebellar cortex, the hippocampus, and in deep grey matter nuclei [8,9,10,11]. In addition to demyelination and oligodendrocyte loss, demyelinating cortical lesions show neuritic transection, neuronal death and reduced presynaptic terminal numbers [8,12]. In progressive MS lesions diffuse pathology is also present in normal appearing white and grey matter, reflected by diffuse axonal injury with profound microglia activation within a background of a global inflammation of the entire brain and the meninges [13]. Interestingly, MRI studies suggest that cortical atrophy may be more closely related to diffuse neurodegeneration in the normal appearing white matter than to the extent of focal white-matter demyelination [14].

In recent decades, better understanding of mechanisms underlying RRMS has led to the development of different disease-modifying therapies, reducing both severity and frequency of new relapses through immune system modulation [15,16]. In contrast, therapeutic options available for progressive MS are comparatively disappointing, and remain a challenge. One possible reason may be lack of knowledge regarding the pathogenic mechanisms driving progressive MS. At present, abnormal tissue findings seen in progressive MS remain poorly represented in experimental animal models.

This review discusses present knowledge on grey matter involvement in progressive MS, as well as the putative mechanisms that can determine the processes of neurodegeneration and neuronal death.

## 2. Grey Matter Changes Observed in MS

### 2.1. Cortical Compromise in MS

**Figure 1:**
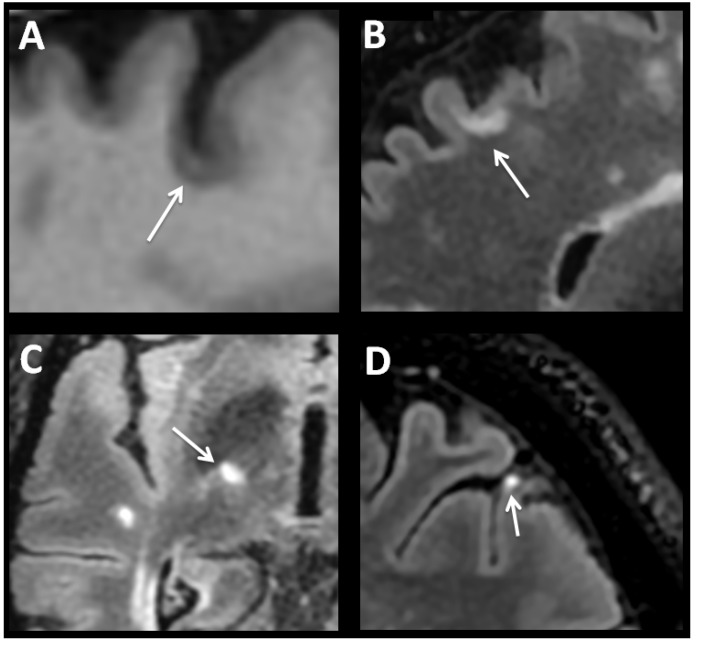
(**A**) Three-dimension sagittal T1-weighted. Hypointense cortical lesion (white arrow). (**B**) Three-dimension sagittal T2-Fluid Attenuated Inversion Recovery (FLAIR). Hyperintense leukocortical lesion (white arrow). (**C**) Axial FLAIR. Subcortical temporal demyelinating plaque and perithalamic internal capsule lesion (white arrow). (**D**) Post-contrast 3D sagittal FLAIR. Focal area of leptomeningeal enhancement (white arrow).

Even though MS was considered early on to be a demyelinating disease of CNS white matter mediated by inflammation, the possibility has been raised in recent years that cortical and deep grey matter demyelination may exceed that of white-matter demyelination, with both postmortem and in vivo studies revealing presence of extensive lesions in grey matter (GM) structures [8,17,18]. Initially articles explained GM compromise as a phenomenon associated exclusively with prolonged disease duration and progressive forms. Recently, however, cortical and deep grey matter lesions in the thalamus, caudate, putamen and cerebellum cortex have been detected during early stages of disease independent of white-matter pathology [19,20,21,22]. Indeed, evidence establishing that grey matter involvement related to disease activity and more aggressive forms is growing [23]. In contrast to other neurodegenerative diseases, it is not known whether cortical atrophy in MS is a more diffuse process or develops instead following distinct anatomical patterns. Cortical regions of the frontal lobe, posterior cingulate, insula and temporal lobes (especially hippocampus) as well as of the cerebellum are by far the most frequent areas affected early on, causing disability progression and cognitive impairment [24]. Recently different patterns of cortical atrophy with or without concomitant white-matter lesions have been described in patients with long-lasting MS. Most of these show a non-random and symmetric distribution, as well as, stronger associations with clinical dysfunction than global cortical atrophy [25]. In CNS tissue samples obtained at autopsy, different cortical lesions have been detected [8,17] in around 60% of the cases, while more recent 7T MRI protocols estimate a frequency above 90% [8,17,26]. Three types of cortical lesions have been reported in MS brain tissue: leukocortical, intracortical and subpial [27]. Leukocortical lesions or type 1 lesions seems to start in the subcortical white matter and extend into the cortex to layers V and VI (Figure 1A,B). Cortical sectors of these lesions showed increased numbers of lymphocytes and microglia/monocytes compared to normal appearing cortex from the same brain or from aged-matched control brains, although numbers of these cells are substantially less abundant than those seen in subcortical white matter [8]. Leukocortical lesions have been detected in patients even during the earliest stages of MS. Intracortical lesions or type 2 lesions are located entirely within the cerebral cortex, are not in direct contact with subcortical white matter or pia mater, and are in general small and perivascular. Finally, subpial lesions or type 3 lesions represent the most abundant type of cortical lesions, and are most prominent during progressive stages. These lesions often show myelin loss in cortical layers I through IV spanning several gyri. On occasion, they can involve all six cortical layers, but rarely invade subcortical white matter, and are mostly associated with meningeal inflammation [17,28,29]. With the exception of loss of myelin, subpial lesions lack most of the other pathological signature findings described in white-matter lesions such as blood-brain-barrier breakdown, as well as immune cells infiltration, perivascular cuffs, astrogliosis, loss of oligodendrocyte progenitor cells, and complement activation. Active tissue damage is also associated with microglial activation [22,30]. However, no correlation has been observed between subpial and white-matter lesion loads [31,32], suggesting subpial demyelination occurs independently of white-matter demyelination. General consensus from autopsy studies would indicate subpial lesions are abundant in progressive stages of MS (both PPMS and SPMS) and rare in MS patients with acute disease or during early stages of RRMS.

### 2.2. Deep Grey Matter (DGM) Structures Changes in MS

Although less well studied, DGM structures involvement is often present together with cortical atrophy, particularly of the thalamus. To date estimation of whole-brain volume has been used most often as a surrogate marker of atrophy in MS, because it is relatively easy to measure. However, there is growing evidence that grey matter volume loss may be more pronounced than that of white matter and be more strongly linked to long-term disability [33,34]. The thalamus may be particularly susceptible to neurodegeneration through different mechanisms, of which two are particularly prominent. First, demyelinating lesions can occur in the thalamus and in perithalamic regions (Figure 1C). Indeed, DGM demyelination can be frequently observed in postmortem MS brain, particularly in the caudate, and in the medial and anterior thalamic nuclei [11]. Histopathologic characterization of the thalamic lesions recapitulates the spectrum of active, chronically demyelinated, lesions observed in the white matter. Similar to changes found in cortical grey matter at autopsy, parenchymal infiltration by T and B cells is limited when compared to levels observed in classic active white-matter lesions. Second, recent work has shown clear patterns of grey matter atrophy in patients with MS that are focused in regions that are strongly connected with diverse neuronal networks [25]. Because DGM structures are extensively connected to cortical grey matter regions, atrophy could also be due to a retrograde event resulting from axonal transection in white-matter tracts projecting from the thalamus, or secondary to trans-synaptic deafferentation of thalamic neurons [11,35]. Interestingly, recent studies have shown that volume loss in DGM over time is faster than in other brain regions across all clinical phenotypes, and drives disability [21,36,37]. Together these studies provide strong evidence that thalamic volume and DGM volume more broadly, are dramatically affected in MS.

## 3. Mechanisms of Neurodegeneration

Different theories have been put forward to explain how progressive MS is triggered. One suggestion is that although brain damage is driven by inflammatory processes similar to those observed during RRMS, during progressive disease stages, a microenvironment is created within the CNS favoring homing and retention of inflammatory cells, ultimately making disease-modifying therapies ineffective [38]. A second possibility is that MS starts out as an inflammatory disease, but after several years a neurodegenerative process independent of inflammatory responses becomes the key mechanism behind disease progression [39]. Finally, MS could be a neurodegenerative disease, with inflammation occurring as a secondary response, amplifying progressive states [40,41]. Clearly, these different mechanisms are not mutually exclusive and could occur in combination. Therefore, in MS neurodegeneration and ultimately progression of disease and chronic disability develop as a result of many different molecular mechanisms. These have been summarized in Table 1.

## 4. Inflammatory Events

Evidence from animal models and immunological studies in MS patients suggests that peripheral immune response targeting the CNS drives the disease process during early phases, whereas immune reactions confined to the CNS dominate later phases of progression [42,43]. The composition of the inflammatory infiltrate within the CNS results from the combination of peripheral immune cells influx, and resident cell activation, particularly of microglial cells, which can change their intrinsic “resting” state in response to prolonged inflammation. Among potential candidates driving inflammation during progressive MS, the role of B cells appears to be prominent. B-cell functions that could be of relevance in progressive MS include: antibody production, increased secretion of pro-inflammatory cytokines, deficient production of regulatory cytokines which impact complement activation and T cell function, as well as antigen presentation and ectopic formation of follicle-like structures [44,45]. Ectopic follicle-like structures are pathological tissue formations resembling tertiary lymph nodes, found in the subarachnoid space of leptomeninges close to inflamed blood vessels (Figure 1D), and also present in other chronic inflammatory diseases [46,47]. They can be induced by follicular T- helper cells cytokine networks acting as positive (i.e., IL-21, and IL-22) and negative (i.e., IL-27) regulators, as well by changes in the stromal networks in connective tissue [48,49]. Composition of these pathologic structures is characterized by aggregates of T and B cells often showing T/B segregation, and development of high endothelial venules, and follicular dendritic cell networks [19,46]. They are capable of sustaining in situ antibody diversification, isotype switching, B-cell differentiation and oligoclonal expansion similar to ectopic germinal centers, which can also support the production of autoreactive plasma cells at the site of local inflammation [48]. These structures co-localize with grey matter lesions and parenchymal infiltrates [50], and are present during different stages of development, ranging from simple T and B-cell clusters to highly organized follicles encapsulated by reticulin lining [51]. Once follicle-like develop, lymphoid chemokines CCL19, CCL21, CXCL12, and CXCL13 are critical for their perpetuation and function, controlling homing recruitment, maturation and antigenic selection of B cells [52], which in turn sustain a high level of humoral response within the CNS independent of peripheral inflammation. This is of particular relevance during progressive MS, when the BBB is intact and contribution to disease activity from entry of peripheral immune cells into the brain is negligible. Antibodies against both myelin antigens and to non-myelin antigens such as neurofascin, neurofilaments and the glial potassium channel KIR 4. 1 has been shown to play an important role in axonal and neuronal damage through complement cascade activation [53,54,55]. In progressive MS cortical demyelination, neurodegeneration and atrophy show positive correlation with diffuse inflammatory infiltrates and lymphoid-follicle structures in leptomeninges, indicating activation of these structures contribute to cortical pathology [2,19,23]. As in other chronic inflammatory diseases follicle-like structures occur in around 40% of SPMS cases [45,56], but are uncommon in PPMS cases. However, it is not known whether follicle-like structures are a typical feature of different disease subtypes from the beginning, or develop as a result of persistent tissue damage and antigen release [20,49]. Notably, meningeal inflammation in SPMS is associated with damage of glial limitans, and a gradient of neuronal loss, which is greater in superficial cortical layers (I-III) nearer the pial surface than in inner cortical layers [23]. These findings suggest cytotoxic factors diffusing from the infiltrated meninges may play a major role in subpial cortical lesions development. Indeed, presence of follicle-like structures in patients with SPMS has been associated with a more severe clinical course, shorter disease duration and earlier death [28,57,58]. Despite this evidence, some studies have reported no substantial perivascular infiltration in pure intracortical lesions found postmortem in patients with longstanding progressive MS [8,17]. These contradictory findings could be due to a reduced sample size, or to insufficient inflammatory activity in the tissue analyzed. Of note, questions remaining regarding neurodegenerative and immunological mechanisms underlying PPMS and SPMS pathology are different. In both cases diffuse meningeal inflammation and cortical neuronal pathology may be significant contributors to clinical progression, suggesting similar pathogenic mechanisms, irrespective of a prior relapsing–remitting course, or the presence of follicle-like structures [59]. Differences observed between both forms of the disease are more quantitative than qualitative in nature [60]. Because serological and epidemiological studies have found an association between B-lymphotropic Epstein–Barr virus (EBV) infection and MS [61], it has been hypothesized that EBV infection of CNS- infiltrating B cells may drive MS pathology [62]. Analysis of postmortem brain tissue from MS patients with different forms of disease, have shown that accumulation of EBV-infected B cells/plasma cells in the meninges and perivascular compartment of white-matter lesions is common and that numbers of EBV-harboring cells correlates with the degree of brain inflammation. Absence of EBV in brain-infiltrating B cells in other inflammatory neurological diseases indicates that homing of EBV-infected B cells to the CNS is specific to MS and not a general phenomenon driven by inflammation [63]. Colonization of cortical lesions has been associated with EBV-encoded small nuclear mRNA (EBER) transcripts in B cells and plasma cells, predominantly expressed during the latent phase of viral infection. Expression of the latency proteins EBNA2 and LMP1, which provide proliferative and prosurvival signals to B cells, in active white-matter lesions and in the meninges in most MS cases, as well as the presence of foci of B-cell proliferation in the MS brain tissue, support a mechanism of EBV-driven B-cell expansion. Ectopic follicle-like structures contained numerous LMP1^+^, but no EBNA2^+^ cells. Meanwhile lytic proteins BZLF1 and BERF1 were found restricted to plasma cells located in active cortical lesions, indicating these structures represent main sites of viral reactivation [63]. Because cells expressing EBNA2 and LMP1 are usually not found in blood, their presence in brain suggests complete disruption of EBV regulation [64]. However, other authors report absence of CNS EBV infection in MS [65]. Interestingly, early lytic EBV antigens elicited CD8-mediated immune responses, triggering strong cytotoxic effects in brain tissue [66]. Indeed, the most active cortical MS lesions are often crowded with CD8^+^ T cells, and contain few B cells o plasma cells, suggesting cortical inflammation correlate with reduction in both B and plasma cell numbers [67]. These observations suggest that EBV reactivation combined with a strong cytotoxic antiviral response mediated by CD8^+^ T cells may drive acute inflammation in both white and grey matter, as well as within the meningeal compartment. CD8^+^ T cells can also recognize specific antigens present on oligodendrocytes, neurons or axons. Once activated, they may be partly responsible for demyelination or axonal/neuronal damage in MS [68,69,70]. Most CD8^+^ T lymphocytes recovered from MS lesions belonged to a few clones [71]. Samples obtained from patients studied longitudinally have shown that certain CD8^+^ T cell clones found in MS patients may persisted over many years in CSF and/or CNS tissue [5,72]. In sharp contrast, the repertoire of CD4^+^ T cells recovered from the CNS in MS patients is heterogeneous [5,71,72]. Overall, these findings reinforce the concept that CD8^+^ T lymphocytes present in the CNS of MS patients are not just bystander cells but are engaged in active immune responses [73]. Axonal damage in white-matter lesions correlates with the number of both CD8^+^ T cells [74] and of activated microglia/macrophages [75] and resident CNS cells which show intense MHC I expression in all types of inflammatory lesions [76]. These observations collectively suggest that in white-matter lesions, CD8^+^ T cells contribute as effector cells causing oligodendrocyte as well as axonal damage. However, there is still controversy over the underlying mechanisms, through which cytotoxic CD8^+^ T lymphocytes harm axons and neurons in MS. Cytotoxic CD8^+^ T lymphocytes release cytokines, such as IFN-γ, and TNF-α, as well as perforin, and granzymes A and B [70,77,78]. IFN-γ for instance, can increase glutamate neurotoxicity and Ca^2+^ influx into neurons through modulation of the IFN-γ/AMPA Glutamate receptor complex [78]. TNF-α on the other hand triggers cell death via the p55 receptor present on neurons [79]. Perforin and granzymes directly damage the cell membrane, causing Na^+^ and Ca^2+^ influx, ultimately leading to energy breakdown and consequent activation of lytic cell enzymes (see below). Granzymes disrupted calcium homeostasis by increasing resting levels, and enhancing IP3-mediated endoplasmic reticulum calcium release. Elevated concentrations of Ca^2+^ are sufficient to activate calcium-dependent death effectors, including caspases [80]. Although perforin did enhance GrB-mediated neurotoxicity, recombinant GrB can itself induce neurotoxicity, independently of perforin [80]. Likewise, interactions between Fas antigen on CD8^+^ cytotoxic T lymphocytes and Fas ligand on neurons triggers Ca ^2+^ release from intracellular storage sites resulting in additional activation of the intracellular caspase cascade causing further axonal/neuronal damage [81].

The role of cytotoxic CD4^+^ T cells in progressive MS has not always been highlighted. However, recent studies demonstrated an increase of this T cell population in late/chronic Experimental Autoimmune Encephalomyelits (EAE) lesions as compared with acute lesions. Moreover, proportions of cytotoxic CD4^+^ T cells were further enriched in the CSF from SPMS patients as compared with corresponding blood samples [82]. These cells arise from repeated antigenic stimulation, after which they lose the co-stimulatory molecule CD28, presenting a cytotoxic phenotype, comparable with NK and CD8^+^ T cells [83]. In addition, CD4^+^CD28^-^ T cells lose their sensitivity to apoptosis induction [84], and are resistant to the suppressive actions of regulatory T cells [85]. Expansion of CD4^+^CD28^-^ T cells is associated with several autoimmune and chronic inflammatory conditions, including MS [86,87], whereas in healthy individuals they are almost undetectable [88]. They have been identified not just in the circulation of patients with chronic inflammatory diseases, but also in target tissues. In MS CD4^+^CD28^-^ T cells are capable of migrating to the CNS mainly through the fractalkine (CX_3_CL1-CX_3_CR1) system. It comes as no surprise that patients who have high numbers of these cells have more severe disease and poor prognosis. Indeed, recently baseline percentage of CD4^+^CD28^-^ T cells was associated with multimodal evoked potential (EP), indicating a link between these cells and disease severity. In addition, the baseline CD4 ^+^CD28^-^ T cells percentage had a prognostic value since it was associated with EP after 3 years and with EP and Expanded Disability Status Scale (EDSS) after 5 years [89]. Notably, in patients with chronic inflammatory disorders it has been shown that CD4^+^CD28^-^ T cells have oligoclonal antigen receptors [90], produce high levels of inflammatory cytokines such as IFN-γ, and GM-CSF, and express cytotoxic molecules (e.g., NKG2D, perforin and Granzyme B), features similar to innate-like T cells, which together could lead to neuronal and axonal loss similar as described by CD8^+^ T cell [68,83]. It remains unclear to date which are the antigens or cues that trigger and/or drive the expansion of CD4^+^CD28^-^ T cells and what stage they acquire cytotoxic activity that contributes to tissue damage and consequent disease progression in MS.

Active demyelination and neurodegeneration have also been linked to microglial activation in early lesions [91]. While in the surveillance state, microglia monitor brain parenchyma detecting danger signals. This state seems to be maintained through a number of interactions with neurons. For example, interactions have been described between CD200-CD200R, CD47-CD172a, and fractalkine-CX3CR1 interactions. As a consequence of brain injury or disease these interactions are lost and resident microglia change their phenotype developing an “activated” state. This change can be induced through several mechanisms including: production of pro-inflammatory cytokines released by Th1 or Th17 T cells, presence of microbial pathogens (PAMPs) recognized by Toll-like receptors (TLRs) or leucine-rich repeat containing receptors (NLRs), release of intracellular components from necrotic or apoptotic cells, as well as presence of heat shock proteins, misfolded proteins (DAMPs) or components of the complement cascade [92]. Microglial activation is not restricted to lesions, but is also diffusely present in normal appearing white and grey matter [13]. In normal appearing white matter (NAWM) for example clustering of activated microglia, so-called microglial nodules, are abundant in areas adjacent to plaques, particularly in patients with progressive MS [93]. Notably, microglia nodules have been associated with damaged axons expressing amyloid precursor protein (APP) accumulation, and changes in neurofilament phosphorylation in the periplaque white matter. Furthermore, direct spatial association has been observed between microglial nodules and axons undergoing Wallerian degeneration [94]. These findings indicate microglial activation is associated with signs of neuronal damage and tissue atrophy strongly suggesting microglial cells contribute to CNS damage in progressive MS.

**Figure 2:**
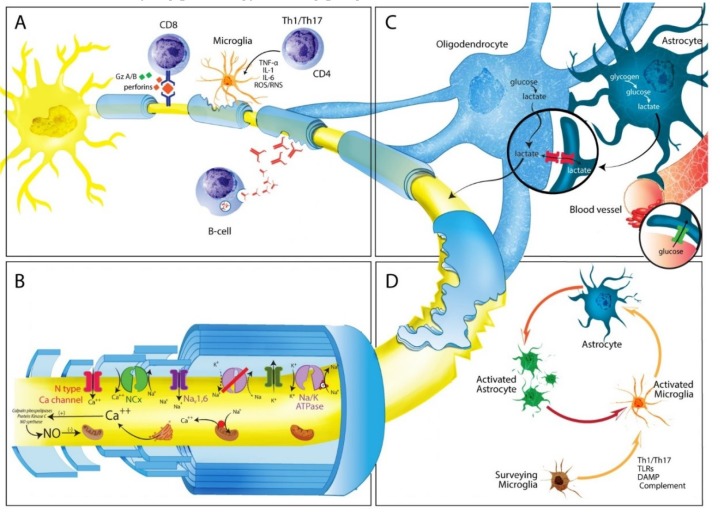
Possible mechanisms involved in MS progression. (**A**) In progressive MS the inflammatory phenomena eventually leading to axonal degeneration and loss are compartmentalized within the CNS. Cellular components are represented by cells that come from the periphery (T and B lymphocytes), as well as by resident CNS cells (microglia cells and astrocytes). B cells can form ectopic follicle-like structures resembling tertiary lymph nodes, producing antibodies against myelin and non-myelin antigens, shown to play an important role in axonal and neuronal damage through complement cascade activation. In turn, CD8^+^ lymphocytes can recognize specific axonal antigens and produce tissue damage through secretion of perforin or granzymes A and B. Autoreactive CD4^+^ Th1 and Th17 lymphocytes can activate microglial cells, which in turn produce pro-inflammatory cytokines (IL-1, IL-6, TNF-α) or oxygen or nitrogen free radicals (ROS/RNS) causing axonal damage and neuronal loss through a bystander mechanism. (**B**) Following demyelination, energy requirements increase due to disruption of paranodal myelin loops. Reduction in neuronal ATP production may lead to failure of the Na^+^/K^+^ pump failure, generating a sustained sodium current, which drives reverse sodium/calcium exchange and accumulation of intra-axonal calcium. This, in turn activates degradative enzymes, including proteases, phospholipases, and calpains, resulting in further neuronal and/or axonal damage as well as impaired ATP production. (**C**) Axonal damage could be cause by poor trophic support. Oligodendrocytes capture glucose from circulation, breaking it down glucose to form pyruvate or lactate, which can enter axons, and be imported by mitochondria for ATP synthesis. An alternative source of energy for axons comes from glycogen stored in astrocytes, which can be transformed into glucose and later into pyruvate or lactate, depending on oxygen availability. (**D**) Several mechanisms cause surveillance microglia activation including Th1 or Th17 T cells; presence of microbial pathogens (PAMPs) recognized by Toll-like receptors (TLRs) or leucin-rich repeat containing receptors (NLRs); release of intracellular components from necrotic or apoptotic cells; presence of heat shock proteins, misfolded proteins (DAMPs), or components of the complement cascade. Once activated they in induce activation and proliferation of astrocytes, leading to astrogliosis.

Damage induced by microglial cells in MS is mediated through different mechanisms (Figure 2A), including secretion of pro-inflammatory cytokines such as IL-1, IL-6, TNF-α, and IFN-γ, phagocytic activity and presentation of antigens to CD4^+^ T cells via MHC Class II molecules [95,96]. Pro-inflammatory cytokines can also induce mitochondrial injury both in neurons and glial cells. In addition, reactive oxygen and nitrogen species (ROS/RNS), produced by microglial cells, cause direct damage to neuron through loss of cytochrome C oxidase (COX1), as well as mitochondrial respiratory chain complex IV activity, leading to mitochondrial dysfunction (see below) [97]. Importantly, release of Fe^2+^ into the extracellular space from injured oligodendrocytes may amplify oxidative damage by generating highly toxic hydroxyl (OH) radicals, from H_2_O_2_. Fe^2+^ uptake by activated microglia determines their fragmentation and degeneration, leading to a second wave of Fe^2+^ release, which can increase susceptibility of surrounding tissues to free radicals-driven axonal and neuronal destruction [98].

Interestingly, cortical demyelinated lesions lack inflammatory lymphocyte or macrophage infiltrates in progressive MS and does not show complement deposition. The majority of phagocytic cells are ramified microglia in close apposition to neurites and neuronal cell bodies [8]. Activated microglia also possesses a puzzling array of neuroprotective functions, including debris phagocytosis and clearance, growth factors production and neuronal-circuit shaping [95]. Distinguish neuroprotective from pro-inflammatory phenotypes remains a challenge when interpreting microglial function.

As previously mentioned, postmortem tissue studies have shown increased microglial numbers and increased activation are associated with variable degrees of axonal/neuritic injury, demyelination, and neuronal loss in cortical grey matter during progressive stages of MS. However, it is as yet unclear how early during the course of MS these degenerative events begin. Future longitudinal in vivo studies linking microglial activation to local cortical atrophy or dysfunction levels as well as to progression of disability in individual subjects should help to improve our understanding of the consequences of cortical pathology at different disease stages. In this context, in vivo positron emission tomography (PET) images of microglia, could clarify the role of activated microglia in MS-related neurodegeneration. Use of a selective translocator protein (TSPO) radioligand 11C-PK11195 allows detection of activated microglia on PET. TSPO is a protein, expressed on the outer mitochondrial membrane of microglial cells, at low levels in the healthy CNS, but up-regulated upon microglial activation [99] making TSPO a sensitive “real-time” marker of activation [100,101]. In non-neoplastic injury to CNS without BBB damage, microglial are the main cell population expressing TSPO. However, blood-derived macrophages, reactive astrocytes, and endothelial cells in the vasculature express TSPO [100,102]. Imaging studies in MS patients using the TSPO radioligand 11C-PK11195 have shown microglial cells activation occurs early on and appears to be linked to disability and brain atrophy [103]. In the NAWM of SPMS patients TSPO binding is significantly increased compared to age-matched healthy controls [102,104]. PET imaging can also be used to differentiate active from inactive chronic lesions. Slowly expanding chronic active lesions are thought to contribute to MS progression. Detection of plaque kinetics in vivo will likely provide new information on underlying pathology driving progression [105].

As in other neurodegenerative disorders, expansion and activation of microglia is the primary mechanism behind astrocytosis (Figure 2D). Although astrocytes survive oxidative stress induced by inflammation and ROS/RNS, they still shown signs of injury, mainly reflected by changes in cell morphology and molecular expression [106]. Scar tissue is composed primarily of astrocytes, however in severe lesions, interaction with other cell types including oligodendrocyte progenitor cells, and fibromeningeal cells also occurs [107]. Several specific molecular and morphologic features have been observed in astrocytes during reactive astrogliosis both in human pathology and animal models [108], of which upregulation of Glial fibrillary acidic protein (GFAP), vimentin, nestin, and the less investigated synemin are hallmarks. Glial scars are evident in tissue from MS patients and mice with EAE and surround areas of demyelination [109]. The purpose of scar formation would appear to be isolation of damaged CNS areas, to prevent spread of tissue destruction. However, glial scar rigidity results in inhibition of both remyelination and axonal regeneration, both negative effects mediated through different mechanisms. Over-secretion of FGF-2 by astrocytes may be detrimental for remyelination, which in turn promotes oliogodendrocytes precursor cells (OPC) proliferation and survival, but prevents maturation [110]. Another molecule that appears to play an important role in preventing OPC maturation is the glycosaminoglycan hyaluronan, which is found throughout the extracellular matrix and CNS white matter [111]. Oligodendrocytes that co-localize with hyaluronan express an immature phenotype, and in vitro treatment of oligodendrocytes precursor cells with hyaluronan in vitro prevents maturation [112]. In addition, astrocytes in injured areas release inhibitory extracellular matrix molecules known as chondroitin sulphate proteoglycans (CSPGs) which can severely in injured areas, affect both cytoskeleton and membrane components of growth cone architecture [113]. CSPGs are a family of molecules characterized by a protein core to which highly sulphated glycosaminoglycan (GAG) chains are attached. Neurocan (secreted) and brevican (cell bound) are the major proteoglycans produced by astrocytes in vitro and both have been shown to inhibit axon growth, following CNS damage [114]. There is clear evidence that CSPGs are produced in excess by astrocytes when they become reactive and that CSPGs inhibitory activity depends on GAG content, as removal of GAG chains from the protein core suppresses CSPG- mediated inhibition [114]. Aside from CSPGs, other less studied inhibitory molecules expressed by astrocytes can suppress axonal growth. Ephrins (EPH) and their receptors for example are secreted by normal astrocytes and increased in MS lesions, inducing axonal growth cone collapse through activation of axon-bound EPH tyrosine-receptor kinase [115].

Likewise, astrocytes as part of the immune system could contribute to disease progression through several mechanisms. First, they can directly affect cell entry to the CNS, via de the BBB, by regulating expression of adhesion molecules, particularly vascular adhesion-molecule-1 (VCAM-1), and intercellular adhesion-molecule-1 (ICAM-1), that bind to lymphocyte receptors very late antigen-4 (VLA4), and lymphocyte function-associated antigen-1 (LFA-1), respectively [116,117]. Second, astrocytes secrete different chemokines such as CCL-2 (MCP-1), CCL5 (RANTES), IP-10 (CXCL10), CXCL12 (SDF-1) and IL-8 (CXCL8), which attract both peripheral immune cells (e.g., T cells, monocytes, and DCs), as well as resident CNS cells (microglia) to lesion sites [118]. In addition, astrocytes can secrete GM-CSF, M-CSF or TGF-β, which can regulate MHC Class II molecule expression by microglia and even their phagocytosis [119]. This could represent the primary mechanism through which astrocytes perpetuate immune-mediated demyelination and neurodegeneration. Recent investigations have demonstrated that in chronic phases of EAE, astrocyte depletion ameliorates disease severity. This deleterious effect of astrocytes on EAE is mediated by preferential expression of 4-galactosyltransferase 5 and 6 (B4GALT5 and B4GALT6) [120]. Notably, B4GALT6 is also expressed by reactive astrocytes in human MS lesions. These enzymes synthesize the signaling molecule lactosylceramide (LacCer), the CNS expression of which is significantly increased during progressive phases of EAE. LacCer promotes astrocyte activation in an autocrine manner [120,121], inducing GM-CSF and CCL2 genes, activating microglia and causing infiltration of monocytes from blood, respectively. Remarkably, inhibition or knockout of B4GALT6 in mice suppresses disease progression, local CNS innate immunity and neurodegeneration in EAE, and interferes with human astrocyte activation in vitro [120].

Third, B-cell-activating factor (BAFF), critical for both B-cell development and survival, as well as for immunoglobulin production, is constitutively expressed by astrocytes in normal CNS. BAFF expression in astrocytes is up-regulated in MS lesions and in EAE affected mice, suggesting astrocytes may contribute to drive B-cell-dependent autoimmunity [122], an important mechanism in disease progression as described above. Finally, an important function of innate immune cells is to act as antigen-presenting cells. However, although astrocytes express MHC Class I and Class II molecules in vitro capable of presenting myelin antigens, their ability to also express co-stimulatory molecules including CD40, CD80, and CD86 challenges this function, making their final effect unclear [123]. Nor is it clear to what degree astrocytes can perform phagocytosis, or process and present antigens, particularly under physiological conditions in vivo [124].

In addition to being part of the immune system, astrocytes contribute to MS progression through production of cytotoxic factors. In rodents, astrocytes stimulated with IL-17 or IFN-γ induce nitric oxide synthase (iNOS) [125]. Likewise, IL-1 as well as combined treatment with TGF-β plus IFN-γ increases the percentage of astrocyte secreted nitric oxide (NO), which is one of the most prominent damage-inducing molecules in neurodegeneration [126,127]. Simultaneously, NO stimulates glutamate release from astrocytes which further increase excitotoxicity [128]. Remarkably, the predominant contribution of NO to excitotoxicity depends on increased superoxide ion O_2_^-^ production, which reacts with NO forming peroxynitrite (ONOO^−^) resulting in neuronal necrosis or apoptosis, depending on its concentration [129]. Furthermore, ONOO^−^ inactivates glutamate transporters in astrocytes, directly damaging myelin, oligodendrocytes, and axons [130]. Decreased uptake of glutamate by astrocyte transporters could also contribute to abnormal levels of extracellular glutamate, which are directly toxic to oligodendrocytes, axons and neurons [131]. Excitotoxicity is caused mainly by sustained activation of glutamate receptors and massive subsequent influx of Ca^2+^ into viable neurons, which in turn results in changes in microtubules and neurofilament phosphorylation, ultimately leading to axon cytoskeleton breakdown (see below) [132].

It is important to note astrocytes have a dual role, not only aiding axonal degeneration, but also creating a permissive environment promoting remyelination [133]. The actual impact of astrocytes on pathogenesis and repair of inflammation therefore, will be dependent on a number of factors, including timing after injury, type of lesion and surrounding microenvironment, as well as interaction with other cell types and factors influencing their activation [134].

## 5. Redistribution of Ion Channels and Axonal Damage

Because pathology findings and number of transected axons correlate with degree of inflammation in MS [7,135], great interest has been focused on neurotoxic products release by the innate immune system, in particular, ROS, RNS, and NO produced by macrophages, microglia, and astrocytes both in MS and EAE [136]. Mitochondria and mitochondrial DNA (mtDNA) are highly susceptible to oxidative injury. ROS and RNS generate mitochondrial enzymes deficit which can be either reversible or irreversible. In MS highly active lesions show diffuse mitochondrial damage, making energy failure the main mechanisms behind functional and structural loss [137]. During progressive MS mitochondrial injury emerges in grey matter, and neuronal cell bodies in deeper layers of the cortex show both impaired mitochondrial activity in the respiratory chain complexes as well as alterations in motor proteins responsible for mitochondria movement from the cell body to axons [96,138]. Axonal transport is essential for neuronal health, and has been implicated in different neurodegenerative conditions. Mitochondria, like other membranous organelles are transported along the axon by two major families of microtubule-based molecular motors, the kinesin family which mediates anterograde transport away from the cell body toward the axon terminal, and cytoplasmic dynein which drives retrograde transport from the distal axon toward the cell body [139]. Notably, in non-demyelinated cortex in progressive MS patients mitochondrial transport deficits, associated with kinesin decrease, preceded structural axons alterations, and morphological changes in mitochondria [140,141]. Additionally, progressive MS neurons in deeper cortical layers present mitochondrias with mtDNA deletions, indicative of an accelerated aging phenotype [138]. Consequences of mitochondrial abnormalities in neuronal cell bodies and axons are two-fold. First, mitochondrial dysfunction results in energy deficiency, which in mild forms will induce functional disturbances, in the absence of structural damage. However, when injury surpasses a certain threshold, energy deficiency will lead to axonal degeneration and cell death [142]. Once a neuronal system has lost it reserves capacity, it is less capable of spontaneous recovery and hence less prone to functional improvement. Second, mitochondrial injury may amplify oxidative stress through release of oxygen radicals, generated as a result of impaired respiratory chain function, establishing a vicious cycle of tissue destruction [143]. Following demyelination, redistribution of certain isoforms of Na^+^ channels (Na_v_ 1.1 and Na_v_ 1.6) along the unmyelinated segment ensues, resulting in increased sodium influx. Early redistribution of Na^+^ channels along denuded axons in white matter of MS plaques and EAE may allow continuation of action potentials in the context of MS recovery of clinical function [144,145]. Interestingly, Na_v_ 1.6, which generates persistent electrical current much larger than those of Na_v_ 1. 2 [146], is co-localized with Na^+^/Ca^2+^ exchanger and with APP, a marker of axonal injury. Conversely, Na_v_ 1. 2 channels may serve an adaptive function with limited ability to sustain high-frequency conduction of action potentials and may contribute to slow depolarization, promoting ectopic firing patterns after demyelination [137]. Slow axonal transport of mitochondria as well as, mitochondrial damage may lead to failure of the Na^+^/K^+^ ATPase pump, generating a persistent sodium current. Na^+^ accumulated in the axoplasm is replaced by Ca^2+^ through a reverse action of the Na^+^/Ca^2+^ exchanger. Increased intra-axonal Ca^2+^ activate a variety of catabolic enzymes including proteases, phospholipases and calpains, ultimately leading to progressive proteolytic degradation of cytoskeletal proteins [147]; (Figure 2B). Moreover, intracellular Ca^2+^ increase results in changes in microtubules and neurofilaments (NF) phosphorylation, ultimately causing cytoskeleton breakdown [132]. Additional deleterious accumulation of Ca^2+^ in axons results from influx via L- and N-type Ca^2+^ channels [148], as well as release from intracellular stores in the axoplasmic reticulum. Abnormal axonal accumulation of Ca^2+^ may also result from glutamate neurotoxicity, which alters intracellular Ca^2+^ homeostasis through a mechanism mediated by axonal AMPA/kainate and metabotropic glutamate receptors, located in the intermodal region of the axons [149]. In addition to Na^+^ channels, others ion channels show parallel adaptive changes to inflammatory stimuli by altering their distribution in neurons as an initial compensatory mechanism, to preserve conductance and axonal integrity. Redistribution of voltage-gated Ca^2+^ channels transient potential receptors melastatin 4 (TRPM4), and acid-sensing ion channels 1 (ASIC1) induce additional overload of Ca^2+^, eliciting further deleterious effects on axons [142].

Abnormal accumulations of NF are a pathological hallmark of many human neurodegenerative disorders. Therefore, neurofilament light chain protein (NfL) together with the neurofilament medium (NfM) and heavy (NfH) subunits, are gaining increasing attention as candidate biomarkers of neuroaxonal injury because they are abundant structural scaffolding proteins of the cytoskeleton, with important roles in axon radial growth and stability, enabling effective nerve conduction velocity, as well as dendritic branching and growth [150]. They are exclusively expressed in neurons and reach abnormal levels as a result of axonal damage and eventual neuronal death. Under normal conditions NF are highly stable within axons and their turnover is low. Pathological processes that cause axonal damage release NF proteins into the CSF and peripheral blood, depending on the extent of damage. Initial studies in MS revealed that CSF levels of NfL were associated with the degree of disease activity and disability [151,152]. Furthermore, CSF levels of NfL, fall as a consequence of disease modifying therapies (DMT), suggesting that NfL can be used to monitor therapeutic efficacy [153,154,155]. However, despite these promising results in MS, a major barrier to widespread adoption of NfL assessment in MS research and clinical practice has been the need for CSF sampling, a problem overcome by use fourth-generation immunoassays, which allow evaluation of serum NfL levels [155]. High serum NfL levels have been associated with disability worsening and relapse status [155,156]. Patients under DMT have lower levels of serum NfL than untreated patients, indicating they are a marker of response to treatment [155]. Notably, a longitudinal study demonstrated patients with increased serum levels of NfL at baseline, independent of MRI variables, experience significantly more brain and spinal cord atrophy over 2 and 5 years of follow-up [156]. Collectively, these observations indicate serum NfL levels can be a useful marker of axonal damage, when applying adequate detection technique.

## 6. Loss of Myelin Trophism Induces Axonal Degeneration

Although myelin is traditionally viewed as a passive insulating structure, recent reports indicate it may exert a more dynamic role. It has become clear that myelin is metabolically active, allowing movement of macromolecules into the periaxonal space with important contributions to axonal health and neuronal survival. Indeed, once myelination is completed, a major task of oligodendrocytes is the provision of energy-rich substrates to axons required for fast axonal transport and propagation of action potentials. Furthermore, bi-directional signaling exists for efficient recruitment of resources, whereby the axons inform their myelinating cells of their metabolic needs proportionally to their activity. The myelin sheath and its subjacent axon should therefore be regarded as a functional unit coupled not only at the morphologic, but also at the metabolic level [157].

Animal studies have shown that oligodendrocytes exert a critical role in maintenance and long-term survival of axons and neurons. Mice mutant of the oligodendrocyte-specific *Plp1* gene, encoding PLP/DM20 a structural component of the myelin sheath, develop progressive axonal CNS degeneration at an older age. However, in this model PLP/DM20 absence has minimal impact on myelination [158]. Likewise, 2’3’ cyclic-nucleotide 3’ phosphodiesterase (CNP) knockout mice develop progressive axonopathy and die prematurely. Interestingly, these mice do not show demyelination at ages when axon degeneration is prominent [159,160]. This is surprising because there is strong evidence that CNP is expressed exclusively by oligodendrocytes. Although the pathology in both mutants is similar, mice deficient in both CNP and PLP develop a more severe axonal phenotype than either single mutant, indicating that each oligodendroglial protein serves a distinct role in supporting myelinated axon function [160]. Axonal pathology preceding axonal degeneration includes altered axonal transport and axonal ovoid formation. These findings are more prominent in paranodal regions, where myelin-axonal communication is most likely to occur, and are highly reminiscent of changes found in CNS tissue from MS patients [158,159]. Studies have also investigated the impact of acute death of oligodendroglia on neuron function and survival. Selective ablation of mature oligodendrocytes induced by diphtheria toxin produces axonal injury characterized by accumulation of non-phosphorylated neurofilaments and APP, without spread of myelin degradation Although some mice exhibited abnormalities in myelin composition, overall myelination was not affected, suggesting axonal injury is not due to demyelination [161]. Taken together, these observations from animal models suggest that the myelin-producing function of oligodendrocytes is not coupled to their role in axon preservation, and that oligodendrocytes themselves are critical for axonal function maintenance and survival in adult life.

During development oligodendrocytes import glucose and lactate to allow rapid myelination synthesize large amounts of lipids. When myelination is complete, oligodendrocytes-derived lactate and piruvate can be taken up by energy-deprived axons for mitochondrial ATP production supporting their energy needs [162]. Several experiments indicate monocarboxylic acid transporters (MCTs) are critical to maintain axonal integrity. Based on sequence homology, 16 MCTs members have been identified, of which only MCT1, 2 and 4 are found in the CNS [163]. As oligodendrocytes accumulate intracellular lactate, this substrate can flow through MCT1 into the periaxonal space, where neurons capture it through MCT2 and metabolize it to supplement energy requirements [162,164]. (Figure 2C). Notably, both genetic and pharmacologic down-regulation of MCT1, which is present almost exclusively in oligodendrocytes, results in axon degeneration and neuronal loss both in vivo and in vitro, without obvious oligodendrocyte damage [165]. Although the observations mentioned above provide strong evidence for a role of oligodendrocytes in directly supplying energy support to axons, other cells including astrocytes may also participate [166]. Astrocytes are essentially the only cells containing glycogen in the adult CNS, and glycogen metabolism followed by glyscolisis provides a source of lactate for other cells [167]. Studies show astrocytes transfer energy metabolites directly to oligodendrocytes, which in turn support neurons and axons metabolism as previously discussed (Figure 2C). Connections between astrcytes and myelinating cells occur via gap junctions formed by connexins (Cx). These gap junctions comprise Cx32 and Cx47 expressed on oligodendrocytes which form heteromeric channels with astrocytes through Cx30 and Cx43 respectively. Double mutant CX32- and Cx43-deficient mice exhibit profound CNS demyelination and axonal injury [168]. Likewise, CX47 and Cx30 double null mice, in which connections between astrocytes and oligodendrcoytes are altered, also developed myelin pathology and severe axonal degeneration [169]. Similarly, loss of Cx43 inhibits glucose delivery to progenitor oligodendrocytes cells and their proliferation, which can in turn influence oligodendrogenesis, and oligodendrocyte metabolic support [170]. Overall these findings provide new insights into the role of oligodendrocytes and astrocytes biology. Identification of bi-directional signaling pathways by which oligodendrocytes influence the axonal metabolism, is highly relevant to understanding MS progression.

## 7. Conclusions and Future Perspectives

Identification of effective therapies for progressive MS remains a priority and a challenge for the MS community. In order to develop new and effective treatment strategies it is necessary to better understand the pathological mechanisms driving disease. Unfortunately, absence of adequate animal models makes identification of potential therapeutic targets even more difficult. In this article we have recapitulated some of the main mechanisms involved in MS progression. Undoubtedly more research will lead to a better understanding of the processes of demyelination/remyelination, as well as of the importance of glial cells in neuronal homeostasis and neuronal degeneration. Clearly, identifying effective therapies for progressive MS would largely be contingent upon a comprehensive understanding of its pathogenesis, animal models incorporating these pathogenic characteristics, novel trial designs including more sensitive outcome measures, and new models of collaboration between physicians and basic science researchers.

## Figures and Tables

**Table 1:** Mechanisms proposed to explain Multiple Sclerosis progression.

Immunological Mechanisms and Effectors	Mechanisms of Neurodegeneration and Axonal Dysfunction
**B Cells**	**Mitochondrial Injury**
Antibody production, Ag presentation, ectopic formation of follicle-like structures Induction of compartmentalized population driving CNS injury, independent of peripheral immune activity. Secretion of IL-6, TNF-α, IL-10, and IL-35: Complement activation and T cell functions EBV-infected B-cell Induce CD8-mediated immune responses against brain tissue	Impaired activity of respiratory chain complexes (I, III and IV) Alterations in mitochondrial molecular motors mtDNA deletions Energy deficiency: failure of Na^+^/K^+^ ATPase, reverse activity of NCX, and excess of intra-axonal Ca^2+^. Amplify oxidative stress. Mediates histotoxic hypoxia, which magnifies energy deficiency
**CD8^+^ cytotoxic T lymphocytes**	**Release of Fe^3+^**
Release of TNF-α: neuronal cell death via p55 receptor; IFN-γ: increased Glutamate neurotoxicity and Ca^2+^ influx; secretion of perforin and granzyme: cellular membrane damage, associated to Na^+^ and Ca^2+^ influx	Iron accumulates with aging. The release of Fe^3+^ from damaged OGD amplifies oxidative injury
Astrocytes *	**Anomalous Distribution of Ion Channels**
Secretion of pro-inflammatory cytokines (IL-1, IL-6, TNF-α), chemokines (CCL-2, CCL-5, IP-10, CXCL-12, IL-8) and BAFF. Blood-brain-barrier breakthrough: action on endothelial cells and tight junctions Activation of microglia: secretion of CXCL-10/CXR3, GM-CSF, M-CSF and TGF-β. Production of Lactosylceramide: induces secretion of CCL2 and GM-CSF Production of ROS, RNS, NO and ONOO-limited Glutamate transporters, increasing Glutamate excitotoxicity Reactive astrogliosis: inhibition of remyelination and axonal regeneration by over-secretion of FGF-2, CSPGs and EPH. Upregulation of purinergic receptors: increased responsiveness to ATP, formation of membrane pores and increased of Ca^2+^ influx Cellular senescence: low level of chronic inflammation, altered Ca^2+^ homeostasis	Redistribution of Na^+^ channels (Na_v_, 1.2, 1.6 and 1.8) along the denuded axon: increased energy demand. Activation of VGCC, ASIC1 and TRPM4 contributes to excess of intra-axonal Ca^2+^ Glutamate excitotoxicity mediates massive influx of Ca^2+^ into neurons Excess of intra-axonal Ca^2+^ stimulates catabolic enzyme systems: leading to proteolytic degradation of cytoskeletal proteins
Microglia *	**Loss of Myelin-Derived Trophic Support and Deficit in Axonal Transport**
Decreased expression of immunosuppressive factors: fractalkine-CX3CR1, and CD200-CD200R. Secretion of pro-inflammatory cytokines: IL-1, IL-6, TNF-α, IFN-γ. Ag presentation of CD4^+^ T cells via Major Histocompatibility Complex (MHC) Class II Oxidative burst: production of ROS and RNS Acquisition of aging phenotype: expression of AGE and RAGE	Alteration of a single myelin protein synthesis (PLP, MGA, or CNP) can cause axonal dysfunction Deficit in axonal transport can reduced expression of kinesins (anterograde transport) and dyneins (retrograde transport)

* Only deleterious mechanisms are presented. Ag: antigen; AGE: Advanced glycation end products; ASIC1: acid-sensing ion channel; BAFF: B-cell-activating factor; CNP: 2′3′ cyclic-nucleotide 3′ phosphodiesterase; CNS: Central Nervous System; CSPGs: chondroitin sulphate proteoglycans; EBV: Epstein–Barr virus; EPH: ephrins; FGF-2: fibroblast growth factor 2; GM-CSF: granulocyte-macrophage-colony stimulating factor; MAG: myelin-associated glycoprotein; M-CSF: macrophage-colony stimulating factor; mtDNA: mitochondrial DNA; NCX: sodium calcium exchanger; NO: nitric oxide; OGD: oligodendrocytes; ONOO^−^: peroxynitrite; PLP: proteolipid-protein; RAGE: AGE receptor; RNS: reactive nitrogen species; ROS: reactive oxygen species; TRPM4: transient potential receptor melastatin 4; VGCC: Voltage-gated Ca^2+^ channel.
